# Cross-sectional metabolic subgroups and 10-year follow-up of cardiometabolic multimorbidity in the UK Biobank

**DOI:** 10.1038/s41598-022-12198-1

**Published:** 2022-05-21

**Authors:** Anwar Mulugeta, Elina Hyppönen, Mika Ala-Korpela, Ville-Petteri Mäkinen

**Affiliations:** 1 Australian Centre for Precision Health, Unit of Clinical and Health Sciences, University of South Australia, Adelaide, Australia; 2 Computational Medicine, Faculty of Medicine, University of Oulu and Biocenter Oulu, Oulu, Finland; 3 Center for Life Course Health Research, Faculty of Medicine, University of Oulu, Oulu, Finland; 4 NMR Metabolomics Laboratory, School of Pharmacy, University of Eastern Finland, Kuopio, Finland; 5 Computational and Systems Biology Program, Precision Medicine Theme, South Australian Health and Medical Research Institute, Adelaide, Australia

**Keywords:** Cardiovascular diseases, Biomarkers, Epidemiology

## Abstract

We assigned 329,908 UK Biobank participants into six subgroups based on a self-organizing map of 51 biochemical measures (blinded for clinical outcomes). The subgroup with the most favorable metabolic traits was chosen as the reference. Hazard ratios (HR) for incident disease were modeled by Cox regression. Enrichment ratios (ER) of incident multi-morbidity versus randomly expected co-occurrence were evaluated by permutation tests; ER is like HR but captures co-occurrence rather than event frequency. The subgroup with high urinary excretion without kidney stress (HR = 1.24) and the subgroup with the highest apolipoprotein B and blood pressure (HR = 1.52) were associated with ischemic heart disease (IHD). The subgroup with kidney stress, high adiposity and inflammation was associated with IHD (HR = 2.11), cancer (HR = 1.29), dementia (HR = 1.70) and mortality (HR = 2.12). The subgroup with high liver enzymes and triglycerides was at risk of diabetes (HR = 15.6). Multimorbidity was enriched in metabolically favorable subgroups (3.4 ≤ ER ≤ 4.0) despite lower disease burden overall; the relative risk of co-occurring disease was higher in the absence of obvious metabolic dysfunction. These results provide synergistic insight into metabolic health and its associations with cardiovascular disease in a large population sample.

## Introduction

The top 10 global causes for death included ischemic heart disease (IHD, 1st), stroke (2nd), dementias (5th), respiratory cancers (6th) and diabetes (7th) according to the Global Health Estimates 2016 report by the WHO. Much of this disease burden is attributed to obesity-associated metabolic dysfunction that increases the risk of cardiometabolic diseases^1^, multiple cancers^2^ and dementia^3^ in ageing individuals. These associations are supported by experimental studies of ageing^4^. There is thus a causal rationale why population subgroups with poor metabolic health bear a higher aggregate burden of multiple chronic diseases later in life.

The predictive power of metabolic profiling has been demonstrated in human populations^5,6^, yet the practical value may be limited for an individual patient^7^. In fact, risk factors for common diseases tend to have small individual impact and *vice versa*^8^, and prediction models for cardiovascular disease have modest performance at individual level^9^ despite clear statistical association at population level. We propose that creating subgroups of metabolically similar individuals may represent a goldilocks solution that combines the robustness of population-wide statistics while retaining easy-to-interpret analogy to observable personal metabolic profiles for more individualised insight compared to traditional epidemiological modelling^10,11^.

In a typical scenario, people with similar profiles are grouped together and the aggregate rates of disease outcomes are compared between the subgroups. For example, we developed subgroups of diabetic complication burden in 2008^12^ and validated them in 2018 with new previously unseen data on clinical outcomes^13^. A recent investigation of body mass and the burden of 400 common diseases in the UK Biobank found clusters with distinct diagnostic profiles, and the authors also provided a comprehensive review of the literature related to biomedical subgrouping^14^. These studies are highly valuable since they produce quantitative descriptors of population health (biomarker profiles) that contain clues on how to reduce adverse long-term outcomes (biological interpretation of the biomarker profiles).

The first aim of this study was to define biologically meaningful metabolic subgroups in a large representative sample of a human population. The second aim was to identify those subgroups that carry the greatest aggregate risk of cardiometabolic and other diseases. To achieve the aims, we used data from the UK Biobank that includes half a million participants, 51 anthropometric and biochemical variables and ten years of follow-up data^15^. We also introduced the self-organizing map (SOM) as a powerful technique to determine metabolic subtypes^11^. Our framework is unique since it combines multi-variate data with expert consensus to infer metabolic subgroups from biochemical profiles while being blinded to clinical diagnoses during model fitting (robust statistics). We interpret these subgroups as prototypical “individuals” that can be used as the basis for targeted public health initiatives, recruitment of representative samples for clinical trials and for identifying synergistic patterns of cardiometabolic risk factors.

## Materials and methods

The UK Biobank is a prospective cohort study of over 500,000 participants aged 37–73 years recruited between 2006 and 2010^15^. UK Biobank has approval from the North West Multi-centre Research Ethics Committee (URL: https://www.ukbiobank.ac.uk/learn-more-about-uk-biobank/about-us/ethics). The participants are volunteers who have provided written informed consent. No personal details were used in this study. Data storage and analyses were conducted according to the material transfer agreement between South Australian Health and Medical Institute and the UK Biobank. This study was designed and implemented according to UK Biobank project plan #29890.

Participants provided baseline information, physical measures and blood and urine samples and information on disease outcomes was obtained through register linkage, including Hospital Episode Statistics (HES), cancer and national death registries. Biochemical measures are described online (URL: https://biobank.ndph.ox.ac.uk/crystal/crystal/docs/serum_biochemistry.pdf). The dataset included in this study comprised 153,731 men and 176,177 women of white British ancestry (Supplementary Figure S1).

The self-organizing map (SOM) is an artificial neural network approach that is designed to facilitate the detection of multi-variable patterns in complex datasets^16^. The result of the analysis is a two-dimensional layout where individuals with similar profiles are close together on the map and thus can be assigned to the same subgroup by visually observable proximity. In this respect, the SOM is a type of clustering analysis, however, in our framework the final step of assigning subgroup labels to individuals is done by human consensus (study authors) rather than by mathematical rules^11^. This is particularly important for population-based datasets such as the UK Biobank that do not have a strong clustered structure due to the broad spectrum of volunteers.

The SOM was trained according to anthropometric and biochemical data; the health outcomes were excluded from the training set to prevent overfitting. The authors were blinded to disease outcomes until after the SOM subgroups were defined. A module-based approach was adopted to avoid collinearity artefacts. First, Spearman correlations were calculated for all pairs of variables. Next, the pairs of variables that were considered collinear (R^2^ > 50%) were collected into a network topology. Lastly, we used an agglomerative network algorithm to define modules of collinear variables^17^ and principal component analysis to collapse each module into a single data column.

The training set was adjusted for age and sex, centered by mean and scaled by standard deviation. The SOM was created with default settings except for smoothness = 2.0 for a more conservative fit. The quality control tests for the SOM are shown in Supplementary Figure S2 (Plots A–L). We verified that every district of the map was populated (sample density ≥ 1293 across the map, Plot A), the model fit was sufficient (residuals below 3 SDs, Plot B) and that the coverage of available data was high (≥ 92% across the map, Plot C). We tested if centering by mean for those under medication affected the map colorings, but we observed no substantial changes in the regional patterns. The map patterns were not confounded by statins (original vs. adjusted LDL, Plots D–F), by anti-hypertensives (systolic BP, Plots G–I) or by diabetic medications (glucose, Plots J–L). To assess the influence of geographical location, we grouped the assessment centers according to latitude into ≤ 51°, 52° and 53°, 54° and ≥ 55°. We did not observe substantial stratification by assessment center location (Supplement Figure S2).

Clinical diagnoses were based on three-character ICD-10 codes (International Classification of Diseases, version 10) from registers of primary care, hospital inpatients, deaths and self-reported medical conditions. Combinations of ICD-10 codes for cardiometabolic diseases are described in Supplementary Table S1. Rheumatoid arthritis, dementia and cancer were included as examples of non-cardiometabolic diseases. Cancer cases were identified using ICD-9 and ICD-10 codes from the cancer registry. The first occurrence of a disease at or before baseline was considered prevalent, new cases after baseline considered incident. Vitality status was obtained from mortality registers censored to 26th April 2020.

Associations with prevalent outcomes were modelled by logistic regression and incident outcomes by Cox regression. Both model types were adjusted for age, sex and assessment center. One subgroup was chosen as the reference and the other subgroups were compared against the reference one-by-one. Cardiometabolic multimorbidity was defined as having at least two out of the four conditions (IHD, stroke, diabetes or hypertension).

Observed multimorbidity was evaluated against simulated null distributions of random co-occurrence of diseases. Firstly, a binary table was created where participants were organized as rows and diseases as columns. To obtain a random sample, the binary columns were randomly shuffled, the aggregate disease tallies were counted for each row and the proportion of rows with a disease tally greater than one was recorded. The process was repeated 10,000 times to create the null distribution. The *P*-value was estimated by comparing the non-shuffled proportion of multimorbidity against the null distribution. Confidence intervals were estimated similarly, except with bootstrapping instead of permutations applied to the binary table. Statistical analyses were conducted with Stata (version 16.0, College Station, TX, StataCorp LP) and R v3.5.0 (URL: https://www.R-project.org/) with the Numero library v1.4^11^.

### Ethics statement

UK Biobank has approval from the North West Multi-centre Research Ethics Committee (URL: https://www.ukbiobank.ac.uk/learn-more-about-uk-biobank/about-us/ethics).

### Consent for publication

UK Biobank participants are volunteers who have provided written informed consent. No personal details were used in this study.

## Results

### Correlation structure between metabolic variables

**Figure 1:**
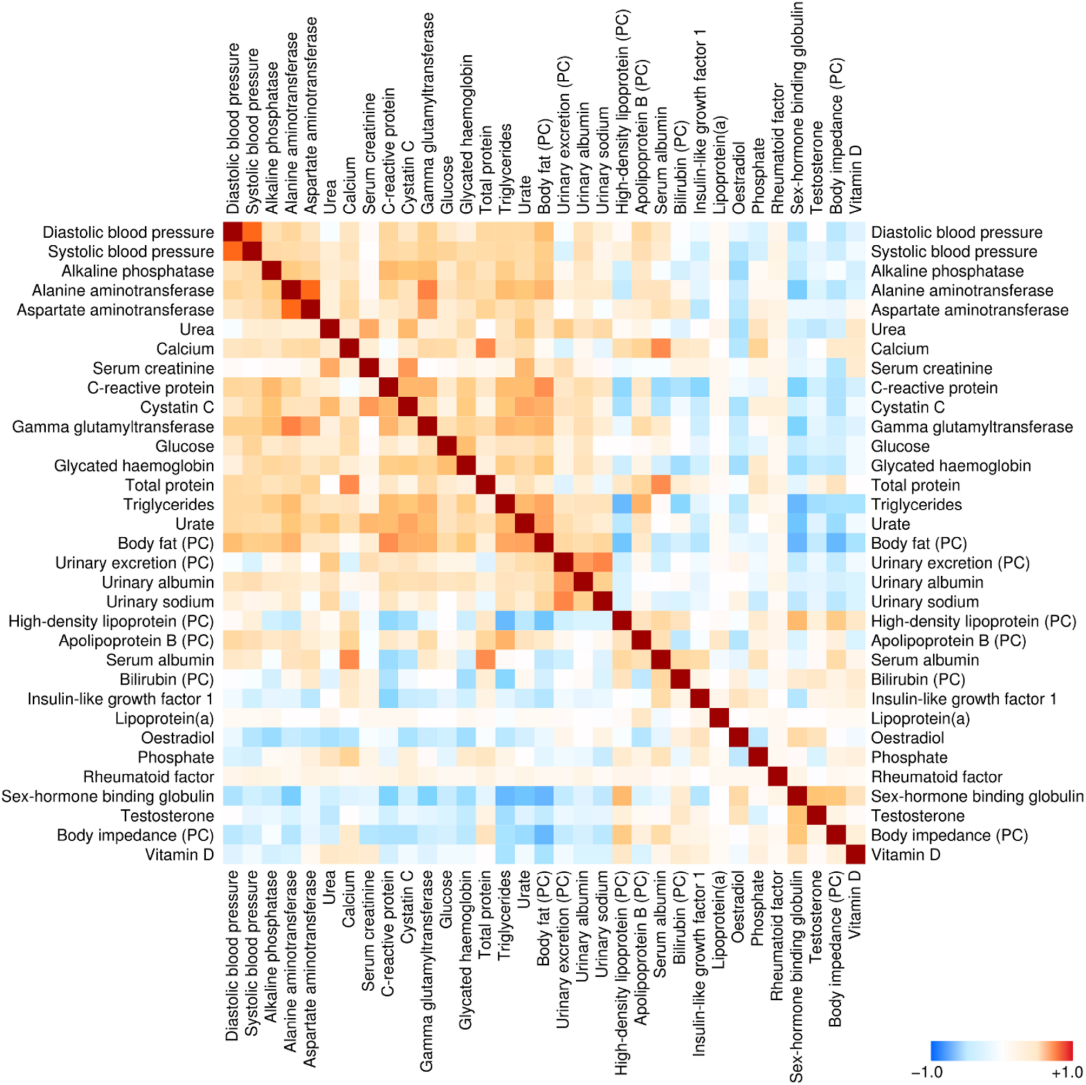
Spearman correlations between anthropometric and biochemical features that comprised the training set for the self-organizing map (adjusted for age and sex). Highly collinear variables were collapsed into the principal component score (PC) prior to correlation analysis.

The characteristics of the study population are listed in Supplementary Table S2. The mean age was 57 years (SD 8 years), most individuals were overweight (BMI mean 27.4 kg/m^2^, SD 4.8 kg/m^2^) and 20,094 (6.1%) individuals died during a mean follow-up of 10.8 years. We investigated 51 metabolic variables (34 biochemical, 15 anthropometric and two blood pressures) that were reduced to 33 SOM inputs based on collinearity (details in Methods, see also Supplementary Figure S3). The final correlation structure is shown in Fig. 1.

### Primer on the self-organizing map

**Figure 2:**
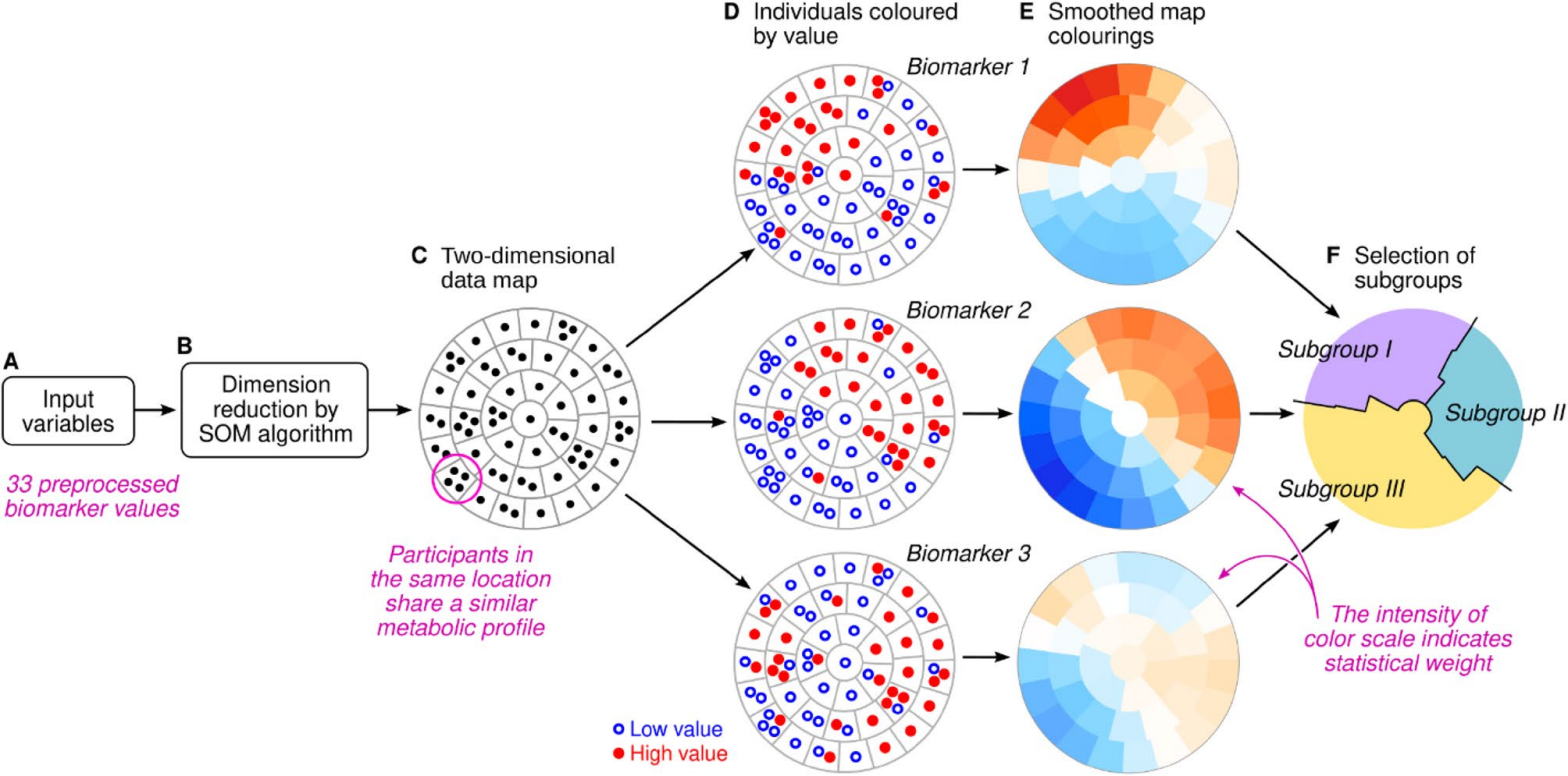
Schematic illustration of the subgrouping procedure. We used the self-organizing map (SOM) algorithm to project high-dimensional data onto a two-dimensional canvas that is divided into districts (**A–C**). The data points can be colored based on the observed values of any variable (**D**). In this study, the statistical weight of regional patterns was encoded in smoothed pseudo-colour representations of the observed values (**E**). The map colorings were used as visual guides to assign map districts and the participants therein into mutually exclusive subgroups (**F**).

The concept of the SOM is illustrated in Fig. 2. Each participant is represented by their individual preprocessed metabolic profile (Fig. 2A, 33 input dimensions). The Kohonen algorithm^16^ is applied to project the high-dimensional input data onto the vertical and horizontal coordinates (two-dimensional layout in Fig. 2B). On the scatter plot, proximity between two participants means that their full multivariable input data are similar as well (Fig. 2C). However, scatter plots are cumbersome for large datasets and difficult to interpret in the absence of distinct clusters. The SOM circumvents these challenges by dividing the plot area into districts. To show statistical patterns, each district is colored according to the average value of a single biomarker or, in the case of morbidity, the local prevalence or incidence of a disease (Fig. 2D, E). The connection between proximity on the canvas and similarity of full profile works the same way on the SOM as it does on a scatter plot. Therefore, selecting a region on the SOM is the same as selecting a subgroup of individuals with mutually similar profiles of input data (Fig. 2F).

The technical details of the SOM have been published previously. In particular, we highlight extensive supplementary documents in four earlier papers that introduce the basic mathematical concepts and discuss the differences between textbook examples of clustered data and the nature of clinical cohort data as the motivation behind the SOM framework^11,17–19^. We also recommend the vignette in the Numero R package (URL: https://cran.r-project.org/web/packages/Numero/vignettes/intro.html) as a practical guide on how to construct a SOM.

### Metabolic subgroups

**Figure 3:**
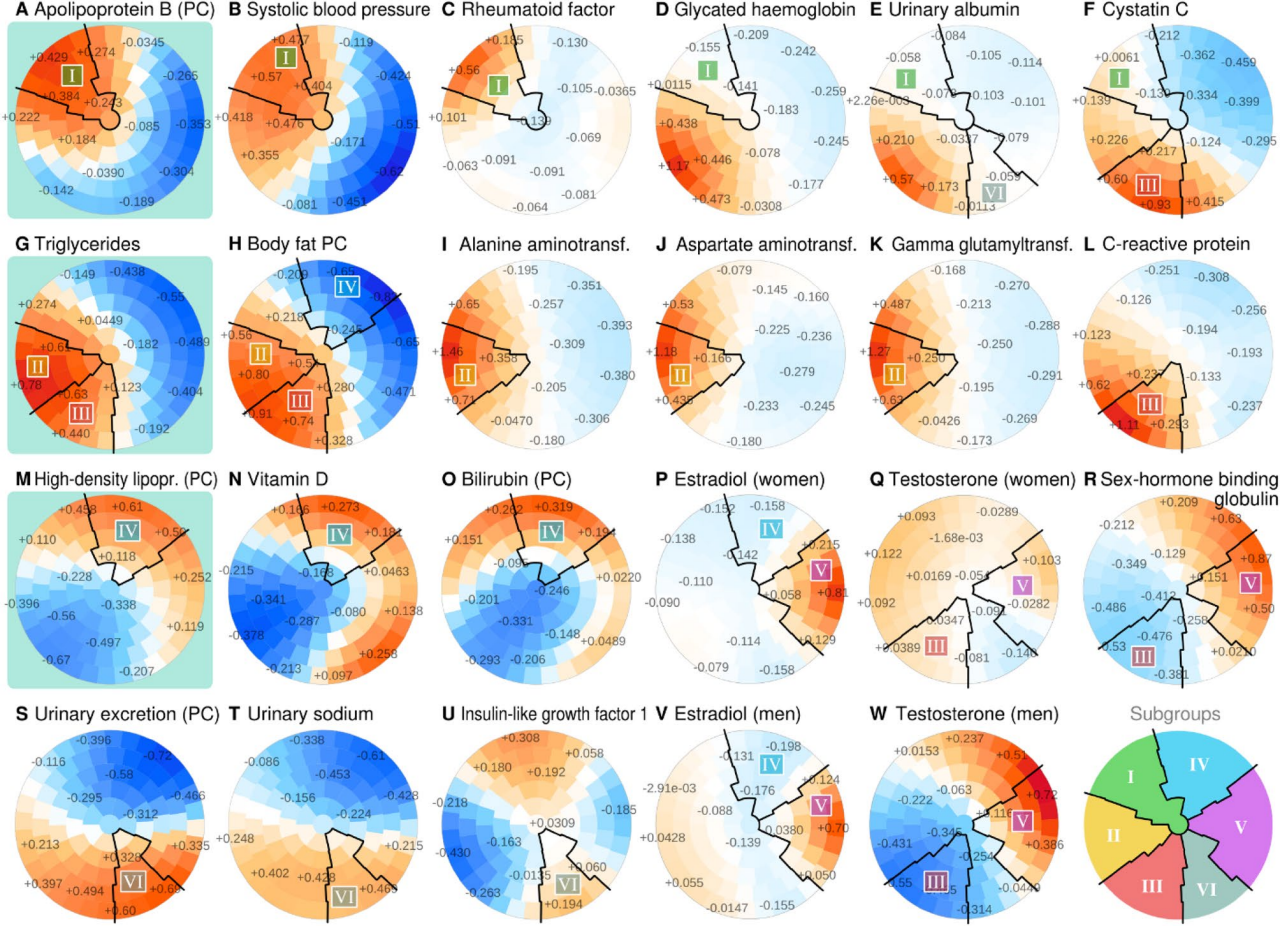
The SOM subgrouping procedure applied to the UK Biobank. In each plot, the same participants reside in the same district. The colors of the districts indicate the regional deviation from the global mean, with color intensity adjusted according to how much the variable contributed to the structure of the map. The numbers on the districts indicate the smoothed mean Z-score of the participants.

IHD is the most common global cause for death^20^ and causally connected to lipoproteins^21^. For this reason, we used the patterns of the apolipoprotein B module, triglycerides and the HDL module as the starting point for subgrouping (Fig. 3A, G, M). We identified map regions that captured the characteristic combinations of features for individuals that had the highest apolipoprotein B score (Subgroup I, top-left part of Fig. 3A–F), elevated triglycerides (Subgroups II and III, bottom-left quadrant of Fig. 3G–L), and the highest HDL score (Subgroup IV, top part of Fig. 3M–P).

Subgroup I was characterized by the combination of high apolipoprotein B score (Fig. 3A), high systolic blood pressure (Fig. 3B), high rheumatoid factor (Fig. 3C) and adequate glycemic control (Fig. 3D). Biomarkers of kidney disease were not elevated (Fig. 3E, F). The second and third subgroups featured elevated triglycerides (Fig. 3G) and high body fat score (Fig. 3H), however, Subgroup II was characterized by high liver enzymes (Fig. 3I–K) whereas Subgroup III had higher C-reactive protein (Fig. 3L). The highest HDL module scores (Subgroup IV) were observed together with the highest vitamin D (Fig. 3N) and bilirubin (Fig. 3O) and low estradiol (Fig. 3P, V). These individuals were the leanest (Fig. 3H).

The highest estradiol values were observed on the left side (Subgroup V, Fig. 3P, V) and Subgroup V also showed the highest testosterone in men (Fig. 3W) and sex-hormone binding globulin for both sexes (Fig. 3R). Sex dimorphism was pronounced; estradiol was fivefold higher in women, and testosterone was tenfold higher in men and we verified that the relative SOM patterns for women under and over the age of 51^22^ were not disrupted by menopause (Supplementary Figure S4). The map area at the bottom (Subgroup VI) was characterized by high urinary excretion biomarkers without albuminuria (Fig. 3E, S, T) and these individuals had higher insulin-like growth factor Z-scores compared to the neighboring Subgroups III and V (Fig. 3U).

**Figure 4:**
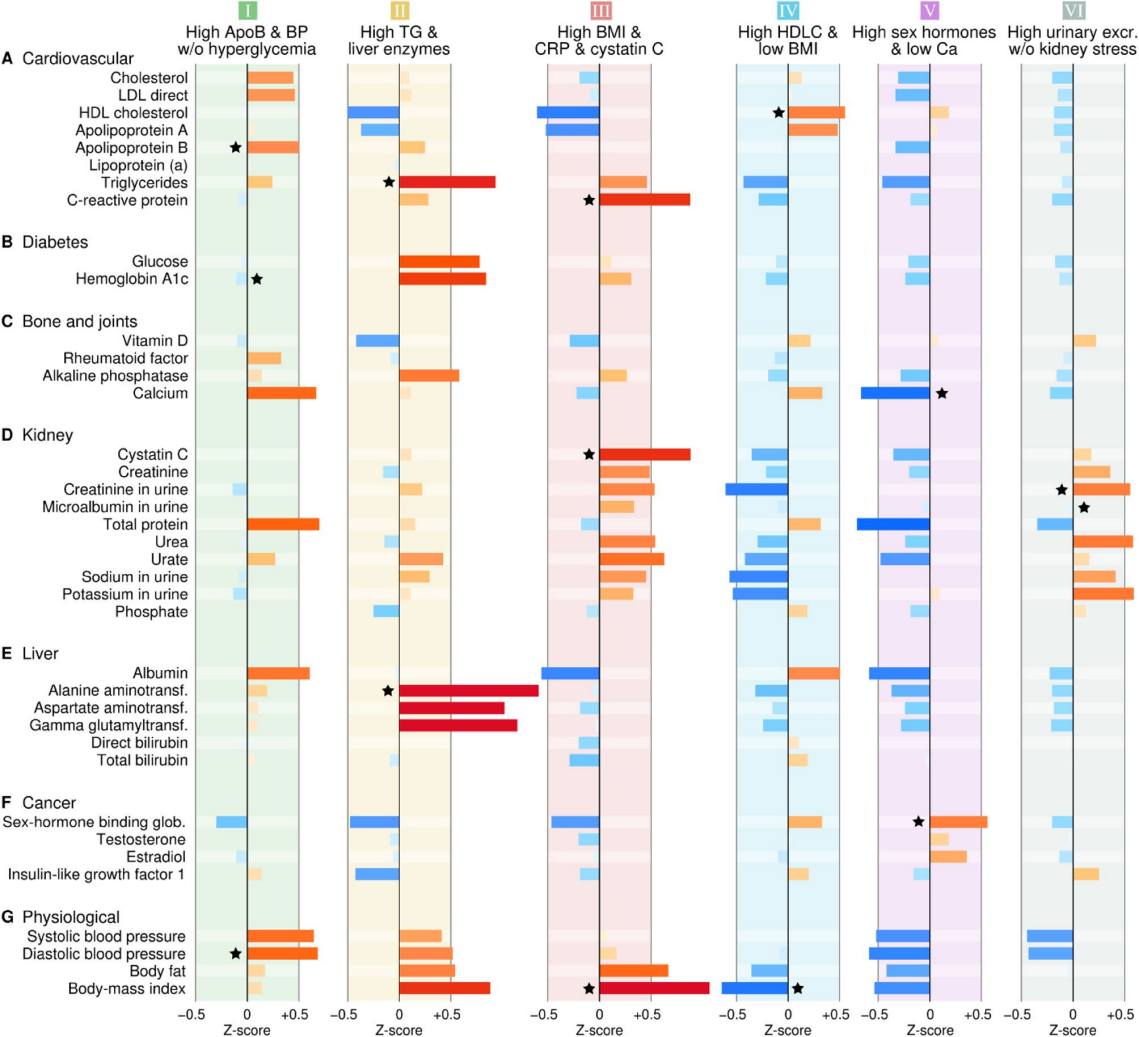
Mean metabolic profiles for SOM subgroups normalized by population SD. The bars are colored according to the direction and magnitude of the deviation from the population mean. The black stars indicate characteristic features that were selected for simplified naming of the subgroups.

Succinct descriptive labels based on selected biomarkers were assigned to the subgroups for easier reading (Fig. 4). Unadjusted map colorings in physical units are included in Supplementary Figures S5 and S6. Numerical descriptions of the subgroups are available in Supplementary Table S3.

### Disease prevalence and incidence by subgroup

**Figure 5:**
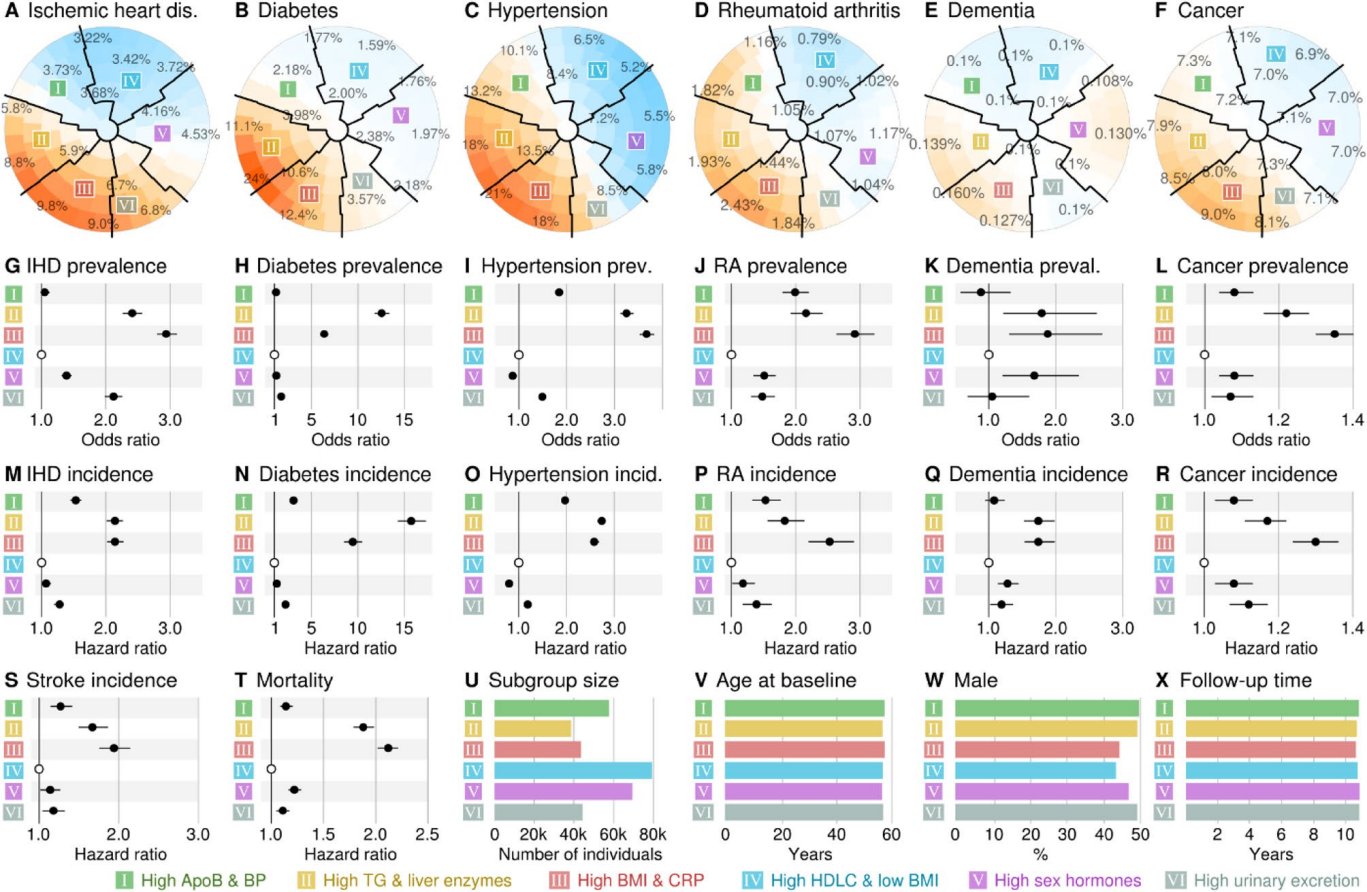
Comparison of morbidity between the SOM subgroups. Percentage of individuals with a disease at baseline across the map districts (**A–F**). Odds ratios for disease prevalence across subgrups based on logistic regression adjusted for age, sex and assessment center (**G–L**). Hazard ratios for incident disease or mortality based on Cox regression adjusted for age, sex and assessment center (**M–T**). Maximum follow-up time available across any clinical end-point (**X**).

The highest prevalence of IHD was observed in Subgroup III (Fig. 5A). Diabetes prevalence varied the most across the map with small percentages for Subgroups IV and V, but substantially higher in Subgroups II and III (Fig. 5B). The pattern for hypertension was close to that of diabetes (Fig. 5C), but there were also individuals in Subgroup I who had hypertension (see also blood pressure in Fig. 4G). The prevalence of rheumatoid arthritis, dementia and cancer was higher in Subgroup III (Fig. 5D–F). Subgroup IV was associated with the lowest overall burden of disease and was chosen as the control subgroup. The subgroups were similar with respect to age, sex and follow-up time (Fig. 5U–X).

Odds and hazard ratios of diseases between the subgroups are shown in Fig. 5G–T and confidence intervals and *P*-values are available in Supplementary Tables S4 and S5. Subgroup III was associated with the highest prevalence of ischemic heart disease (7.5%, OR = 2.9), hypertension (19.3%, OR = 3.7), rheumatoid arthritis (2.3%, OR = 2.9) and cancer (9.1%, OR = 1.4). High incidence was observed for IHD (9.6 per 1000 person years, HR = 2.1) and the highest incidence for rheumatoid arthritis (1.6, HR = 2.53), cancer (12.8, HR = 1.3), stroke (2.6, HR = 1.9) and mortality (13.4, HR = 2.1).

The prevalence of diabetes was the highest in Subgroup II at 16.7% (OR = 12.6) and the incidence was 14.3 per 1000 person years (HR = 15.8). The incidence of ischemic heart disease in Subgroup II was the same as in Subgroup III (9.6 vs. 9.7, *P* > 0.05). There were no differences in the prevalence of dementia (0.13% vs. 0.14%, *P* > 0.05) or the incidence of dementia (1.4 vs. 1.5, *P* > 0.05) between Subgroups II and III.

### Metabolic syndrome and multimorbidity

**Figure 6:**
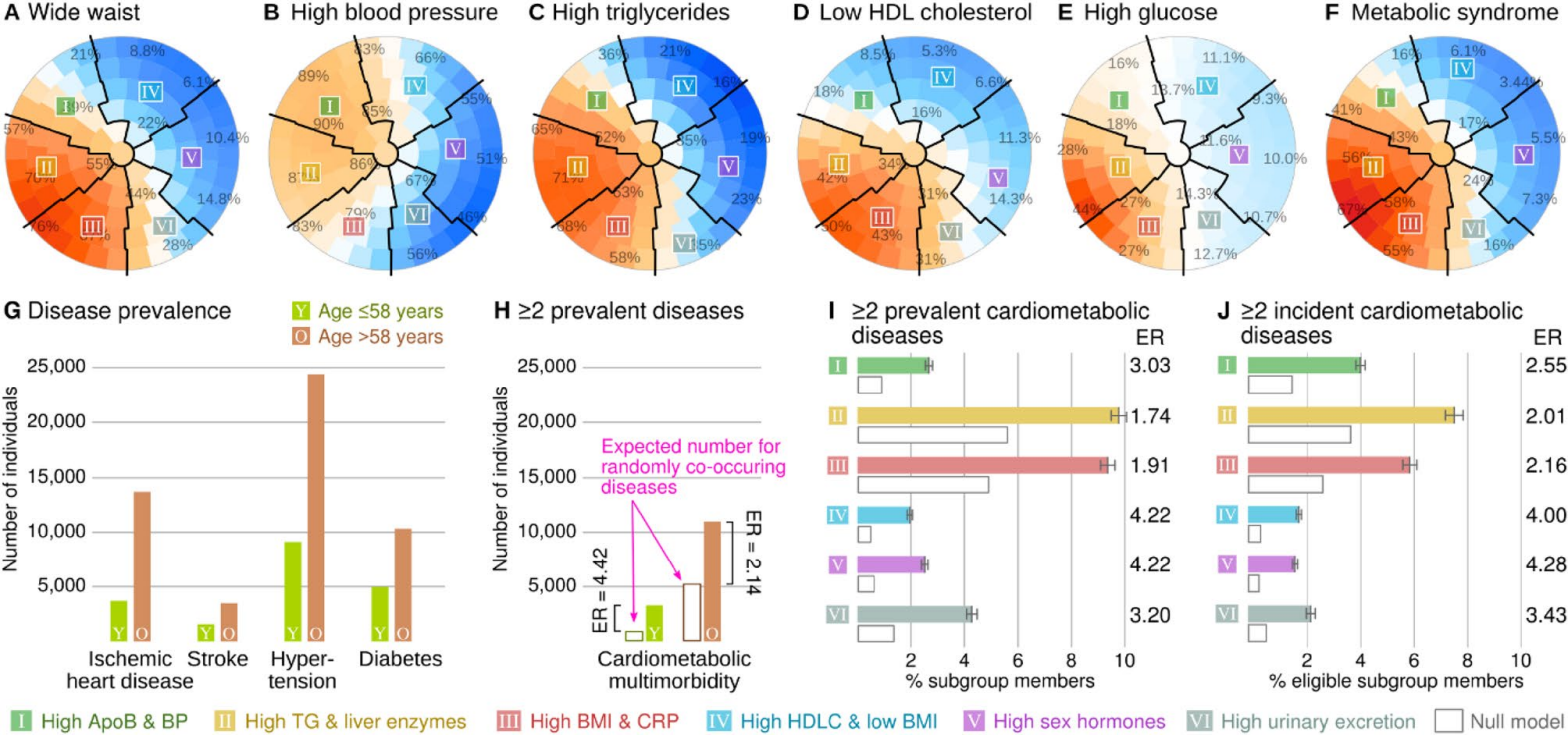
The metabolic syndrome (MetS) and multimorbidity. MetS was defined according to the NCEP ATP III criteria that include five components (**A–E**, the percentages in the plots indicate the proportion of individuals that satisfy a criterion) and subsequent binary classification for those with ≥ 3 points (**F**). The participants were divided into those with age ≤ 58 (*N* = 167,337 or 50.7%) and those with age > 58 (*N* = 162,571 or 49.3%) to create two equally sized age strata (**G**). The null model represents the number of multimorbid cases if the co-occurrence of diseases was random. Bars for subgroups include 95% confidence intervals (**H–J**).

The metabolic syndrome (MetS) was developed to capture synergistic features associated with high cardiovascular risk^23,24^. The SOM patterns for MetS classification (NCEP ATP III) are shown in Fig. 6A–F and numerical results are available in Supplementary Table S6. High MetS prevalence was observed in Subgroup II (64.2%) and Subgroup III (57.8%) and the lowest in Subgroup IV (5.7%).

The MetS combines risk factors, but we also investigated the combination of established morbidities. The burden of multimorbidity depends on the frequencies of the diseases in the population: if two diseases become more frequent, the random chance of having both increases. For example, younger individuals have fewer diseases compared to older individuals (Fig. 6G, split by the median age of 58 years). This difference in disease frequencies leads to a difference in multimorbidity by mathematics alone (the null model, see Methods). However, the observed excess beyond the null model (i.e. enrichment) was greater in younger individuals (Fig. 6H), which means that having one cardiometabolic disease as a young person increases the probability of having another disease more than it would for an older person.

The highest frequency of multimorbidity was observed in Subgroups II (prevalence 9.8%, incidence 7.7%) and III (prevalence 9.4%, incidence 6.1%) and the lowest in Subgroups IV (2.0%, 1.9%) and V (2.5%, 1.8%). We defined the enrichment ratio (ER) as the ratio between the observed number of individuals with ≥ 2 diseases versus the number predicted by the null model. Multimorbidity was enriched in all subgroups (Fig. 6D, E and Supplementary Tables S7 and S8), with the highest ratios observed in Subgroups IV (prevalent ER = 4.22, incident ER = 4.00), and the lowest in Subgroup II (prevalent ER = 1.74, incident ER = 2.01).

## Discussion

Metabolic dysfunction is inextricably linked with ageing demographics and the global obesity pandemic and comes with potentially grave health implications for populations and individuals alike^1–3^. To understand the phenomenon better, we introduced data-driven metabolic subgrouping of the UK Biobank as a model of metabolic diversity (the first aim of the study) and investigated subgroup-specific prevalence and incidence of multiple clinical outcomes (the second aim of the study).

We defined six metabolic subgroups based on the SOM of the UK Biobank. The first three subgroups captured the patterns of classical IHD risk factors and the obesity pandemic (Subgroups I-III). The liver-associated Subgroup II was predictive of diabetes and IHD, which fits with the concept of fatty and insulin resistant liver as a key player in VLDL-HDL dyslipidemia, insulin resistance and type 2 diabetes^25,26^. The inflammatory and kidney stressed Subgroup III was associated with the highest mortality and overall chronic morbidity (including IHD). This pattern is also compatible with the literature^27,28^. The distinction between the liver and kidney is a notable biological insight from the SOM analysis—for example, the popular definitions of the MetS do not capture the liver-kidney spectrum^24^.

We identified a subgroup with elevated sex hormones (Subgroup V). These individuals had a low burden of diabetes and morbidity, which fits the Rotterdam Study^29^ and other evidence on insulin resistance^30^. Yet the Rotterdam study also reported that high estradiol in women may indicate increased diabetes risk. Furthermore, we observed multi-fold variation in absolute levels between men, women, young and old that may confound disease associations, as also noted by other studies^31,32^. Longitudinal studies with multiple time points of hormones may be necessary to understand how hormonal levels indicate and predict metabolic dysfunction.

Subgroup VI was characterized by elevated serum urea, elevated serum and urine creatinine and high urinary electrolytes. There was no clear indication of kidney stress nor high morbidity. The biochemical pattern is compatible with the expected effects of habitual high-protein diet^33^. Subgroup VI may also capture a haemodynamic or a fluid balance aspect of metabolic health^34^. Incidental circumstances during sample collection is another possibility: as there is only one biochemical time point, acute illness or other stressors before the baseline visit may have confounded systemic metabolism and resulted in atypical findings for multiple affected and correlated biomarkers.

Obesity and unfavourable lifestyle are risk factors for multimorbidity^1,35^. However, the previous studies did not consider the confounding increase in co-occurrence when the frequency of diseases increases. We observed a synergistic enrichment for cardiometabolic multimorbidity in all subgroups. The most likely explanation is intertwined etiology, partly due to pleiotropic genetic variants and environmental exposures and partly due to secondary effects between the diseases themselves such as the mechanical stress on the vasculature from hypertension^36^ or toxicity from excessive glycation in diabetes^37^. Another explanation could be diagnostic procedures: if one disease is detected, it is easier to look for and establish the presence of another.

Multimorbidity enrichment was pronounced in the metabolically favorable Subgroups IV and V despite them having lower disease burden overall. The paradoxical finding means that the relative risk of co-occurring cardiometabolic disease was higher in the absence of obvious metabolic abnormality. The pattern may reflect genetic and environmental susceptibility that is independent of the typical cardiovascular risk factors but nevertheless pleiotropic to cardiometabolic diseases^38^. The same pattern may also arise from survival bias as people who are simultaneously affected by metabolic dysfunction and multiple morbidities tend to perish younger^39^.

The statistical link between metabolic dysfunction and cardiovascular disease is strong on the population level but this does not necessarily translate to accurate prediction of individual events^8^, indeed, most cardiovascular risk models show modest predictive ability^9^. For this reason, we envisage the SOM to occupy the inter-mediate space where we can leverage the aggregated statistics over subgroups while interpreting the results as stereotypical individuals that represent meaningful biological phenotypes. Specifically, human observers have visual access to every single variable and its patterns when making the decisions on subgroup boundaries. It is also easy for human observers to verify which subgroup profile matches their own since the profiles are expressible in physical measurement units. Therefore, the SOM model is directly applicable to real-world people and only one SOM is necessary to describe the burden of multiple common disease, as seen in Figs. 5 and 6. Yet a subgroup contains multiple individuals, which enables the calculation of prevalence and incidence rates as subpopulation risk estimates. Indeed, propensity scoring is already used in this manner to identify pools of representative cases within health informatics systems^40^. However, these methods are often presented as black boxes and thus lack the biological context that the SOM colorings can provide. The SOM lets a group of scientists to “see” the data through the statistically standardized colorings in a way no other tool can, and use that information to create a consensus on how to split the population into subgroups that make biological, medical, economic and societal sense.

### Limitations

Due to the large sample size, the statistical robustness is high in this study but we urge caution when generalizing the findings of this study to other cohorts, to other ethnicities or to populations of different circumstances. Furthermore, the results are dependent on the selection of available biomarkers and different laboratory panels may produce different subgroups (note also a previously published comprehensive risk factor screening^41^). We also note that the statistical accuracy of population-based data is insufficient to develop a machine learning model for a clinically robust predictive test^8,9^. The UK Biobank recruited volunteers only, thus people with less opportunity to participate due to low socio-economic status or poor health may be under-represented, however, the disease associations are compatible with other cohorts^42^. Ageing affects metabolism, but the SOM was constructed from cross-sectional data and adjusted for age, thus we are unable to provide information on longitudinal metabolic trajectories and the metabolic subgroups should not be interpreted as part of a temporal sequence.

## Conclusions

The SOM subtypes provided a descriptive framework of how combinations of multiple risk factors are associated with diverging cardiometabolic disease outcomes within a population. The new information is useful for the development of targeted interventions for specific subgroups; potential applications include phenotypically guided trials of new treatments where participants are selected based on their full phenotypic profile (e.g. cardiovascular drug trials designed for persons with inflammatory kidney stress vs. persons with diabetogenic liver stress). Such designs will provide more targeted information on the exact type of patient who will benefit the most from the treatment. We also see potential to adopt metabolic profiles as a new approach to assess the health and aggregate disease burden in a population. For example, subtype prevalences can provide phenotypically specific information on how changes in environmental risk factors influence the aggregate disease burden in different segments of the population over time.

## Supplementary Information

Supplementary Information 1.

Supplementary Information 2.

Supplementary Information 3.

Supplementary Information 4.

Supplementary Information 5.

## Data Availability

The UK Biobank data are publicly available (https://www.ukbiobank.ac.uk/). This study was designed and implemented according to project plan #29890.

## Abbreviations

IHD: Ischemic heart disease
SOM: Self-organizing map
HR: Hazard ratio
WHO: World Health Organization
HES: Hospital episode statistics
ICD: International classification of diseases
BMI: Body mass index
SD: Standard deviation
HDL: High-density lipoprotein
OR: Odds ratio
MetS: Metabolic syndrome
NCEP ATP: National cholesterol education program adult treatment panel
VLDL: Very-low-density lipoprotein
