# Supplementary material for: Cross-sectional metabolic subgroups and 10-year follow-up of cardiometabolic multimorbidity in the UK Biobank
Source: Sci Rep. 2022 May 21;12:8590. doi: 10.1038/s41598-022-12198-1 (PMC9124207; doi:10.1038/s41598-022-12198-1)

Supplement Figure: Map colorings for men

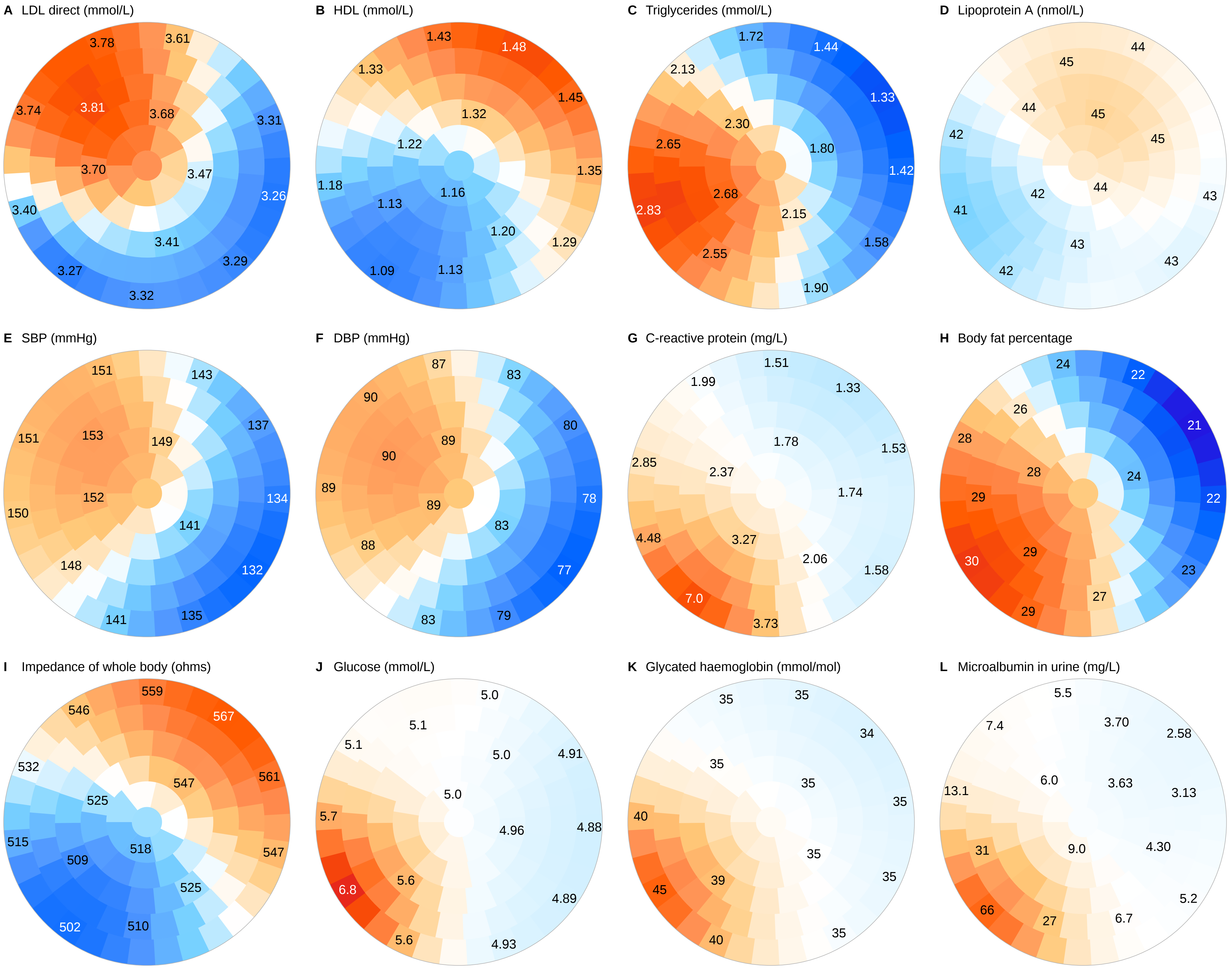

M Sodium in urine (mmol/L)

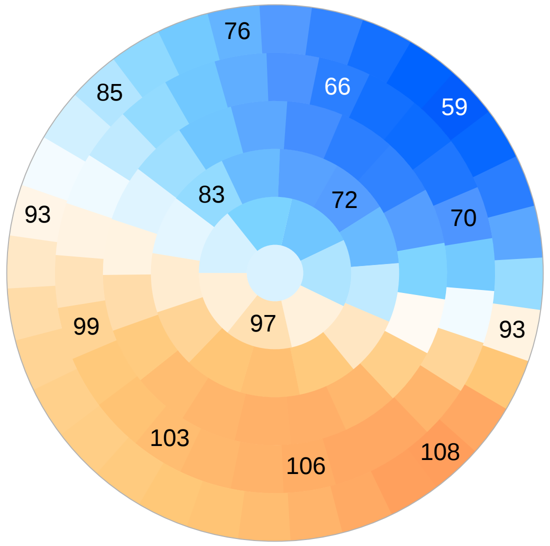

N Creatinine (l’mol/L)

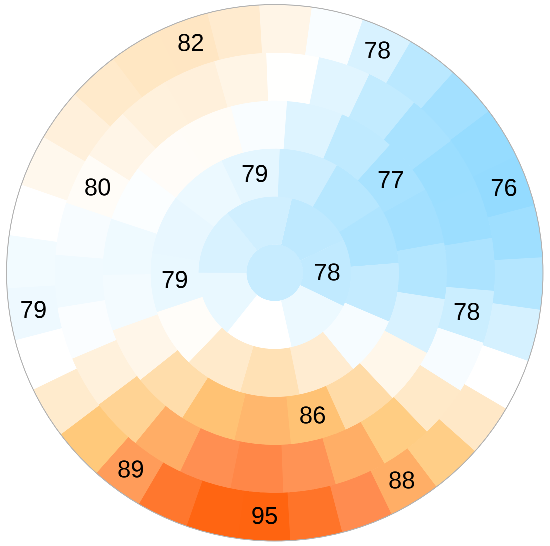

O Cystatin C (mg/L)

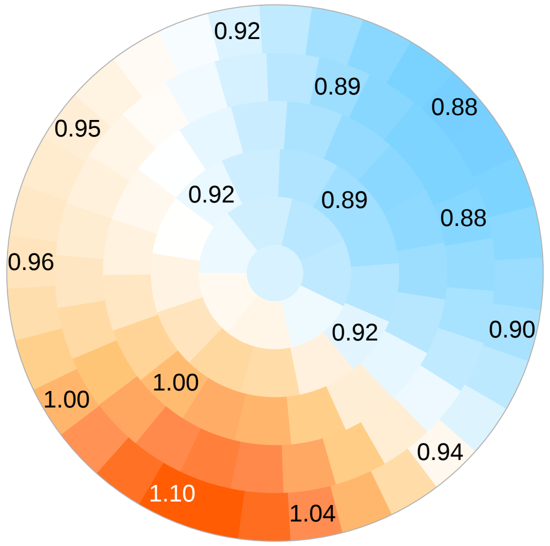

P Total protein (g/L)

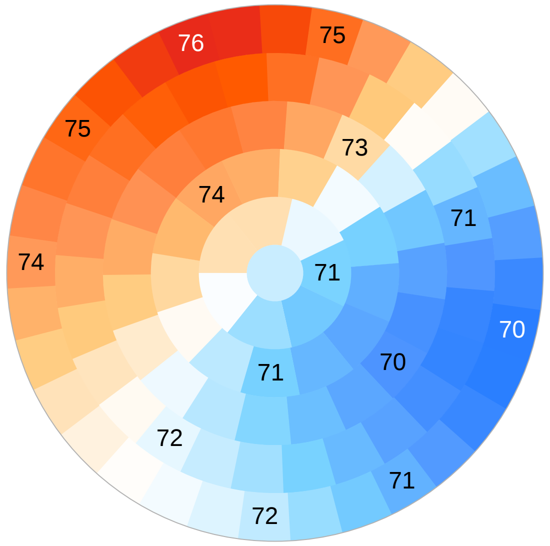

Q Urate (l’mol/L)

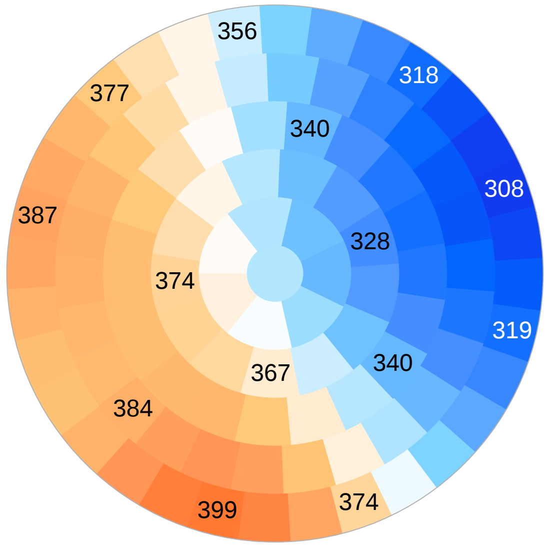

R Urea (mmol/L)

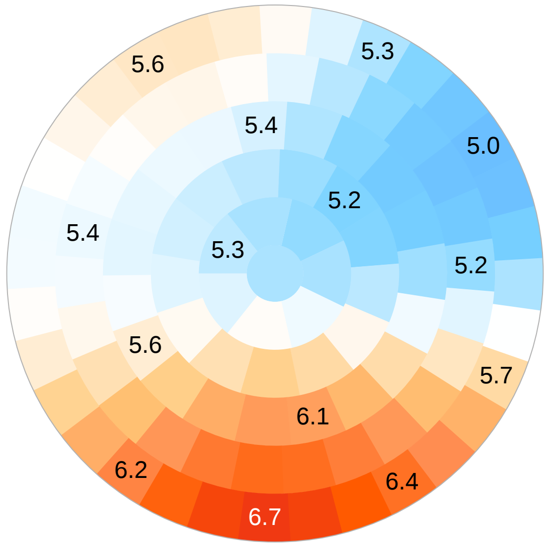

S Phosphate (mmol/L)

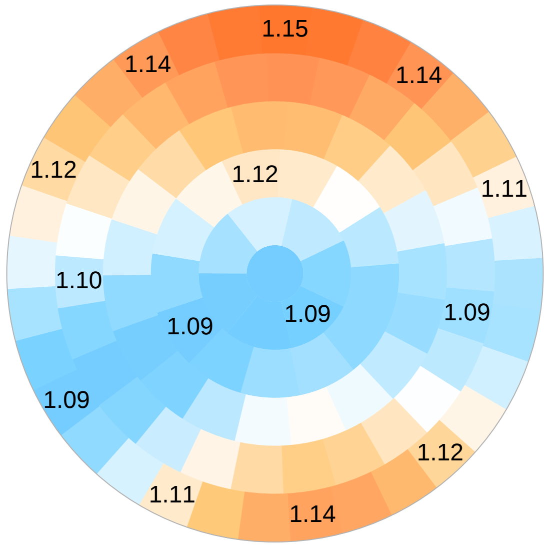

T Testosterone (nmol/L)

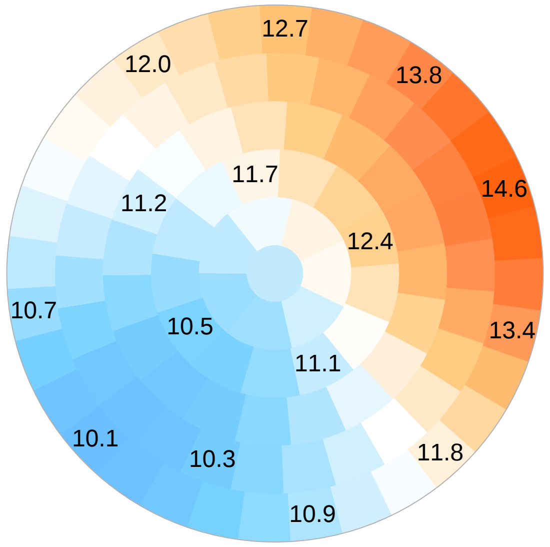

U Oestradiol (pmol/L)

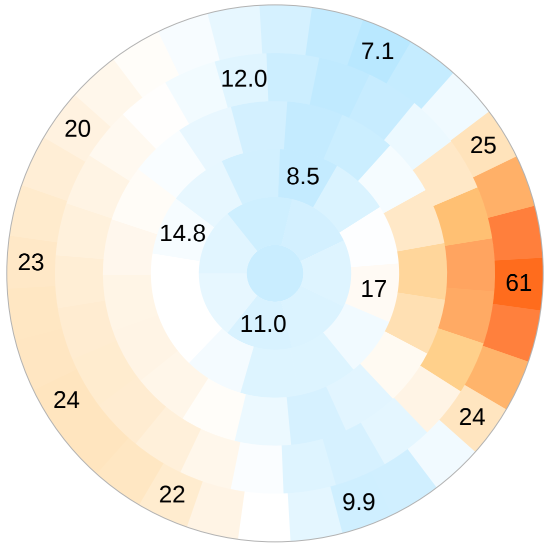

V IGF-1 (nmol/L)

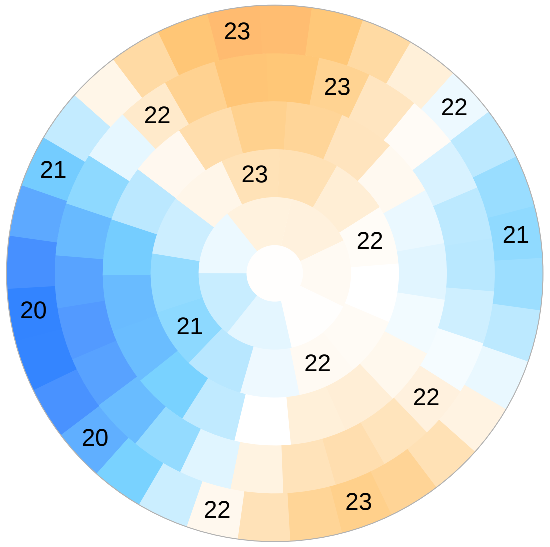

W SHBG (nmol/L)

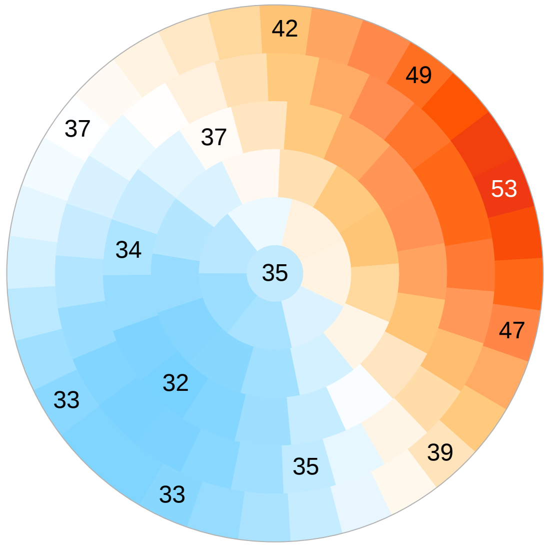

X Calcium (mmol/L)

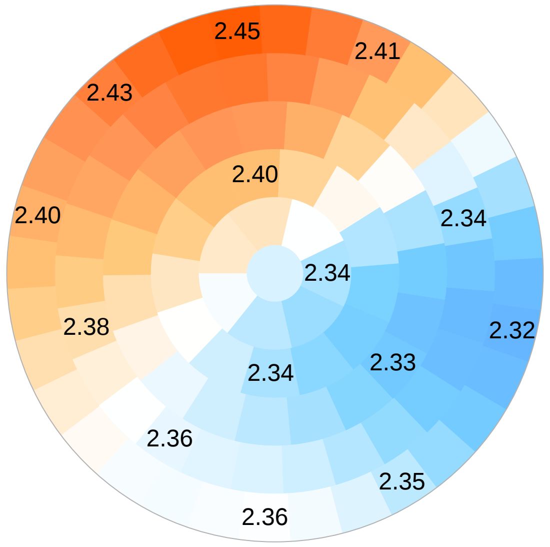

Y Vitamin D (nmol/L)

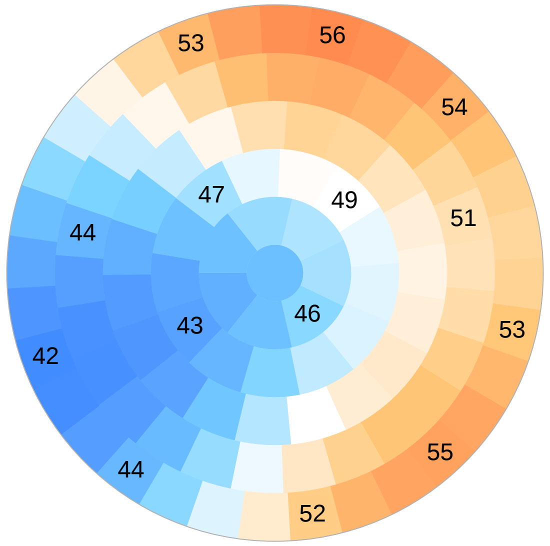

Z Alkaline phosphatase (U/L)

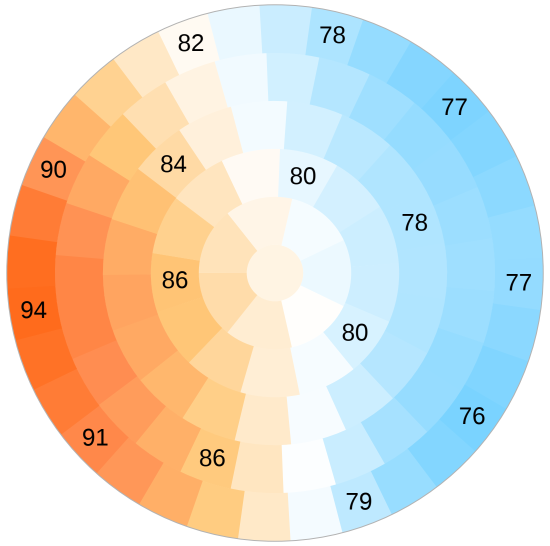

AA Rheumatoid factor (IU/ml)

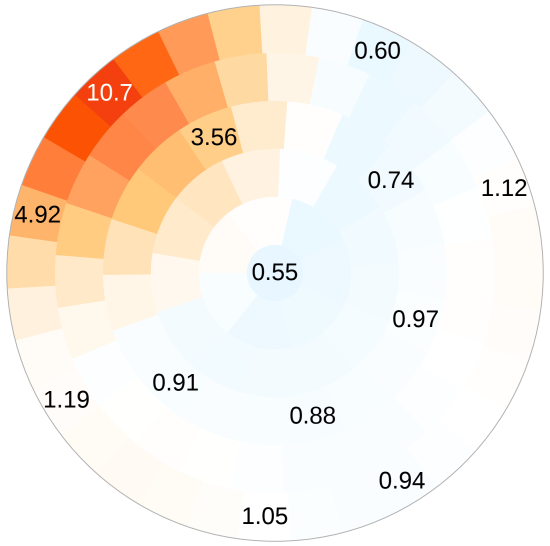

AB Albumin (g/L)

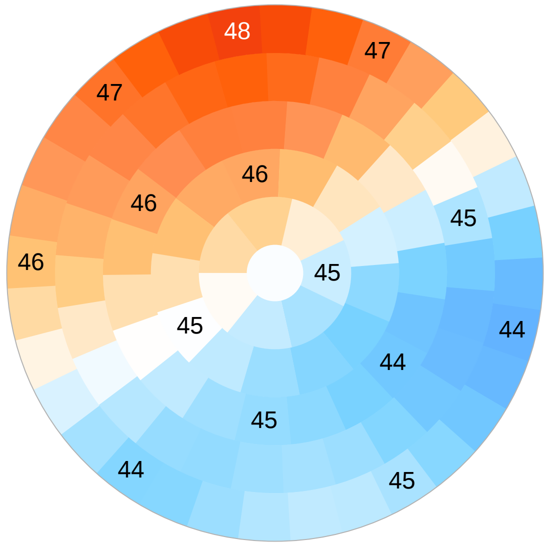

AC Gamma glutamyltransferase (U/L)

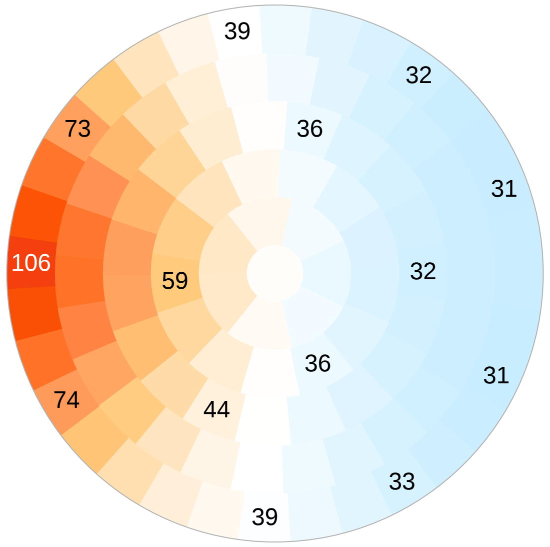

AD Alanine aminotransferase (U/L)

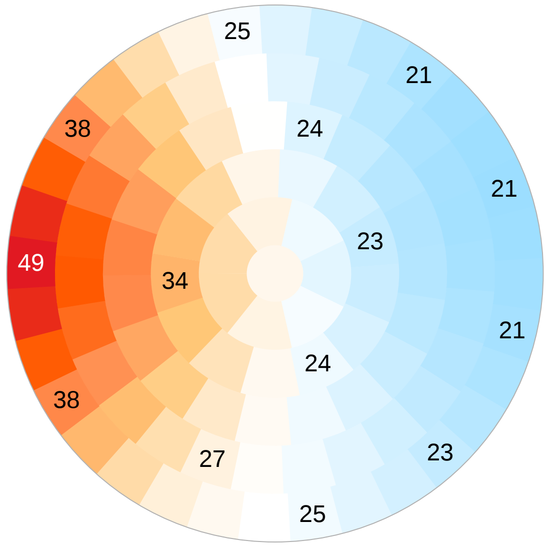

AE Aspartate aminotransferase (U/L)

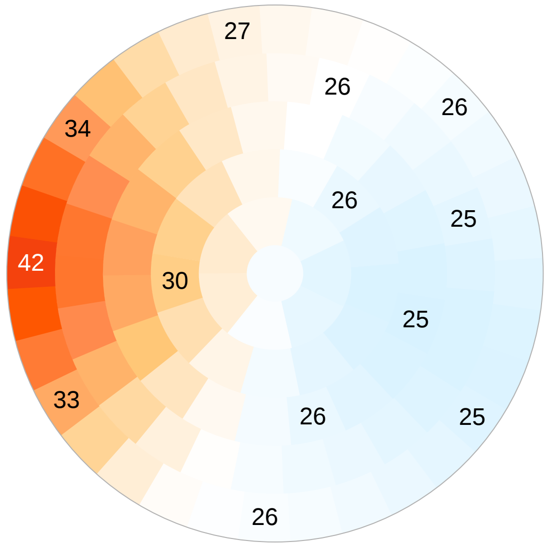

AF Total bilirubin ľmol/L

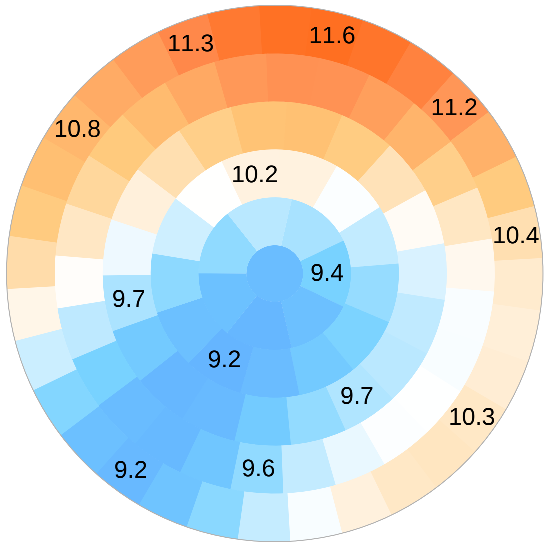

Supplement: Supplementary file 2 — Supplementary Information 2. [file 41598_2022_12198_MOESM2_ESM.pdf]
