# Supplementary material for: Cross-sectional metabolic subgroups and 10-year follow-up of cardiometabolic multimorbidity in the UK Biobank
Source: Sci Rep. 2022 May 21;12:8590. doi: 10.1038/s41598-022-12198-1 (PMC9124207; doi:10.1038/s41598-022-12198-1)

Supplement Figure: Map colorings for women

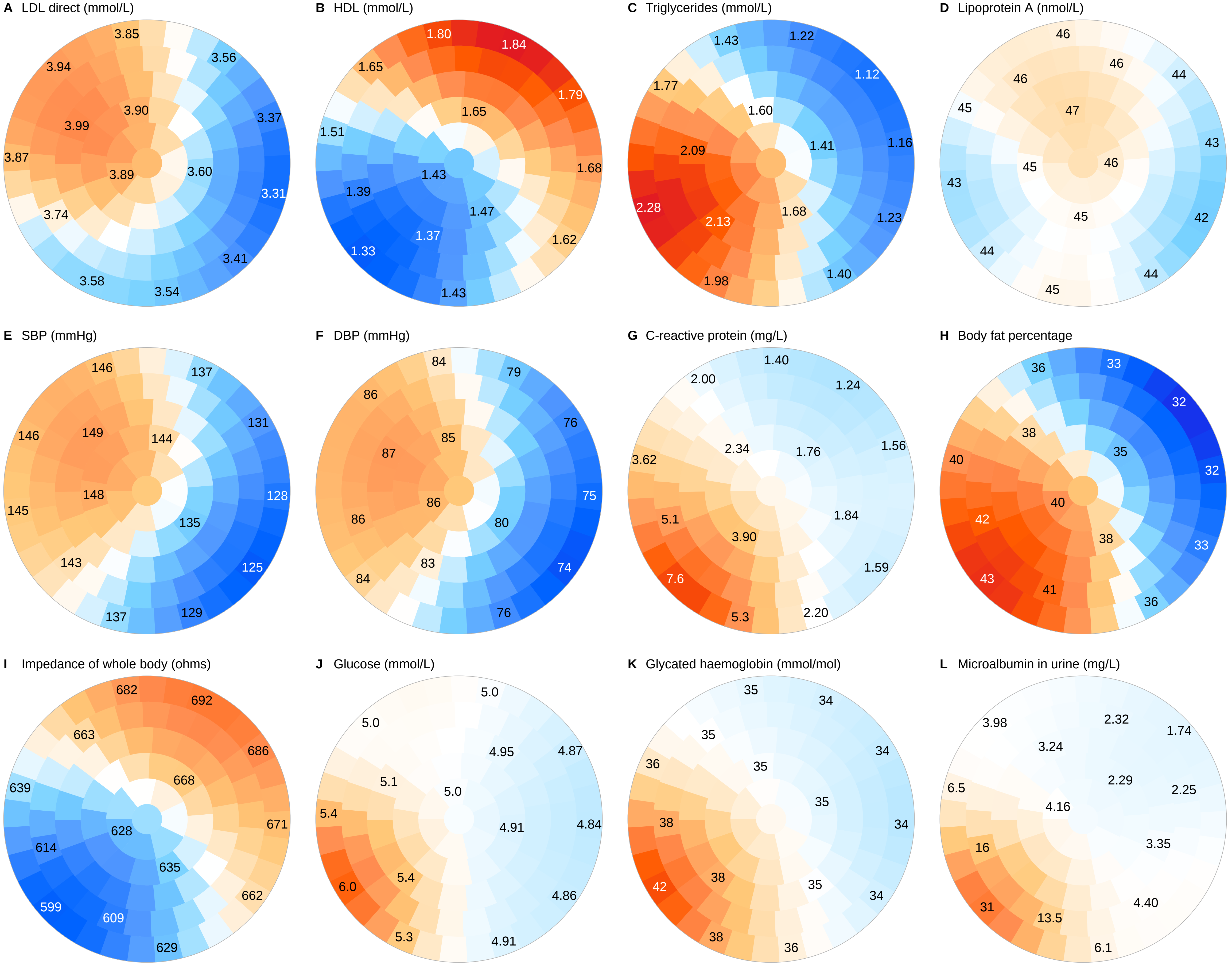

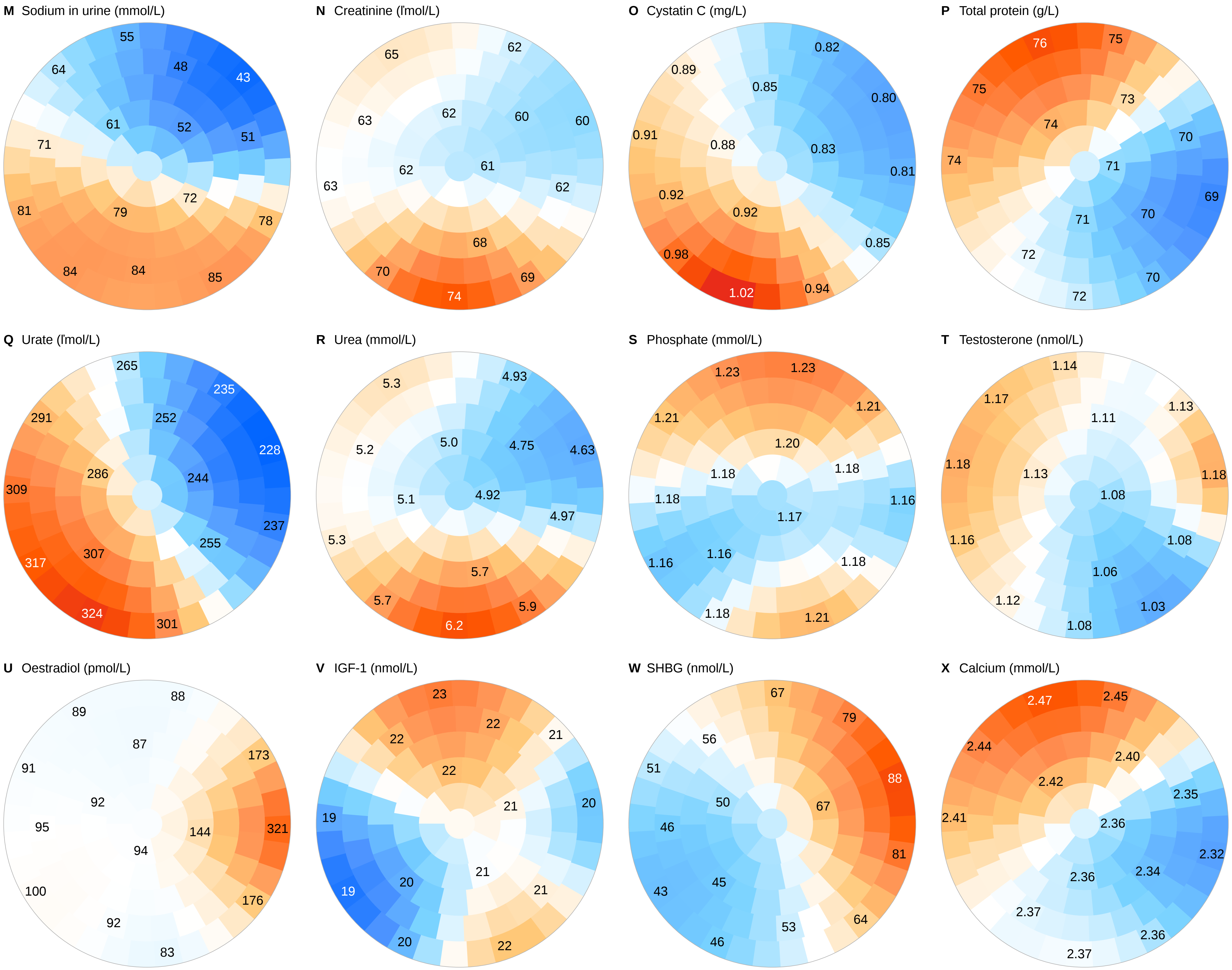
M Sodium in urine (mmol/L)
55 64 48 43 61 52 51 71 72 81 79 78 84 84 85
N Creatinine (Ïmol/L)
65 62 63 62 60 60 62 61 63 62 68 70 74 69
O Cystatin C (mg/L)
0.82 0.89 0.85 0.80 0.91 0.88 0.83 0.81 0.92 0.92 0.85 0.98 1.02 0.94
P Total protein (g/L)
76 75 75 73 74 70 74 71 69 70 71 72 70 72
Q Urate (Ïmol/L)
265 235 291 252 228 286 244 309 237 255 307 317 324 301
R Urea (mmol/L)
5.3 4.93 5.2 5.0 4.75 4.63 5.1 4.92 4.97 5.3 5.7 5.7 5.9 6.2
S Phosphate (mmol/L)
1.23 1.23 1.21 1.21 1.20 1.18 1.18 1.18 1.16 1.17 1.16 1.16 1.18 1.18 1.21
T Testosterone (nmol/L)
1.14 1.17 1.13 1.11 1.18 1.18 1.13 1.08 1.16 1.08 1.06 1.12 1.03 1.08
U Oestradiol (pmol/L)
88 89 87 91 173 92 95 144 321 94 100 176 92 83
V IGF-1 (nmol/L)
23 22 22 21 22 21 20 19 20 21 19 21 20 20 22
W SHBG (nmol/L)
67 79 56 51 88 50 67 46 81 43 45 64 53 46
X Calcium (mmol/L)
2.47 2.45 2.44 2.40 2.42 2.35 2.41 2.36 2.32 2.36 2.34 2.37 2.36 2.37

Y Vitamin D (nmol/L)

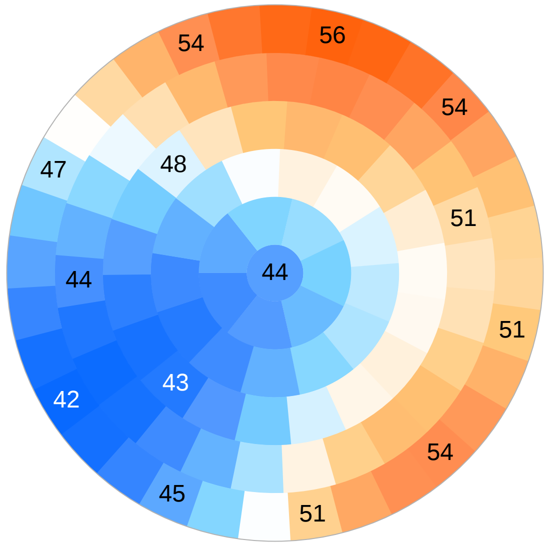

Z Alkaline phosphatase (U/L)

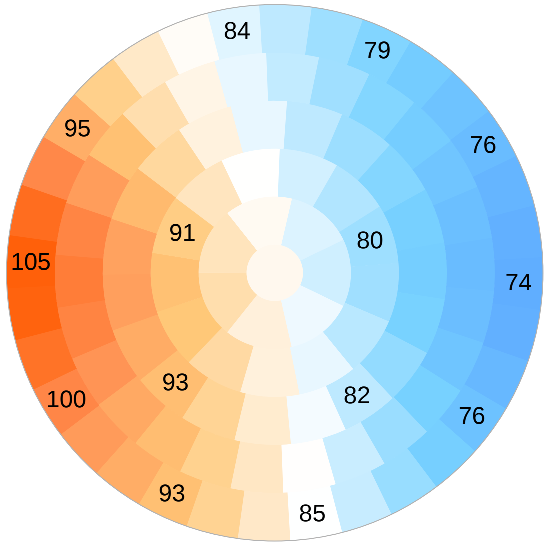

AA Rheumatoid factor (IU/ml)

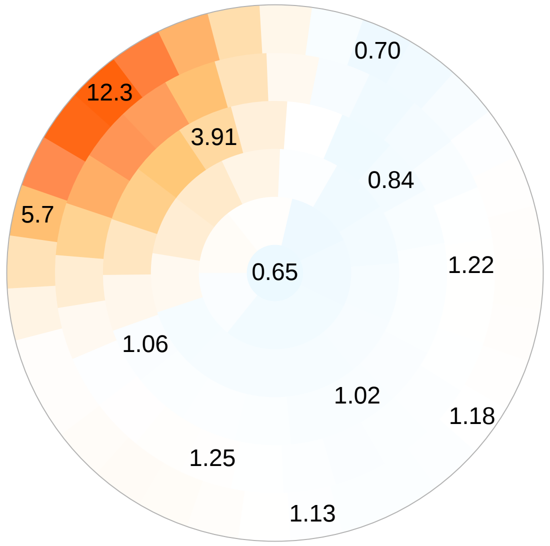

AB Albumin (g/L)

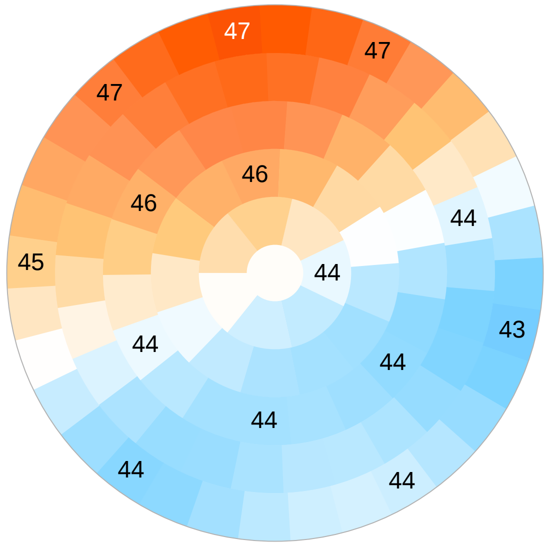

AC Gamma glutamyltransferase (U/L)

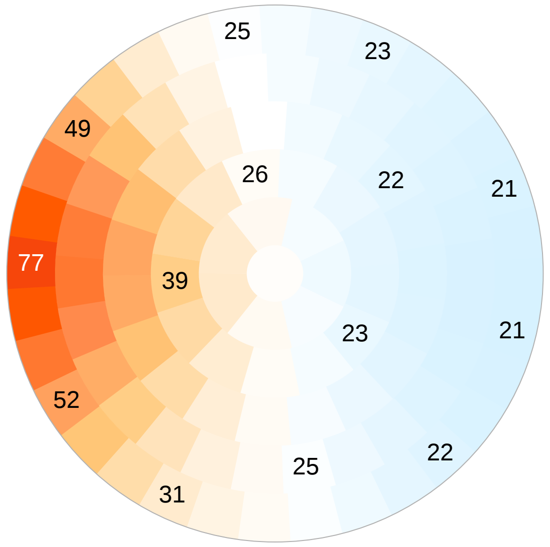

AD Alanine aminotransferase (U/L)

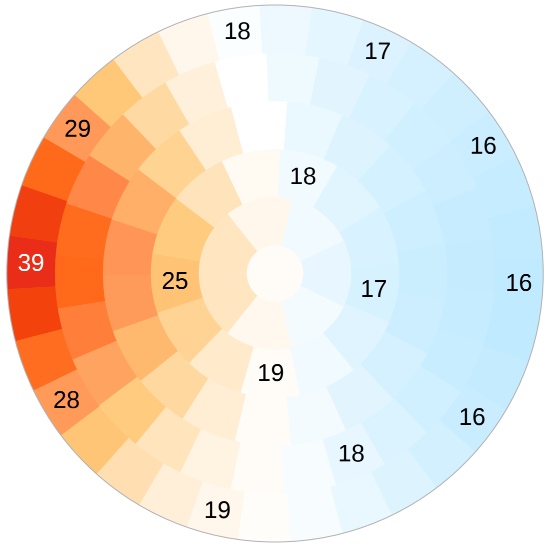

AE Aspartate aminotransferase (U/L)

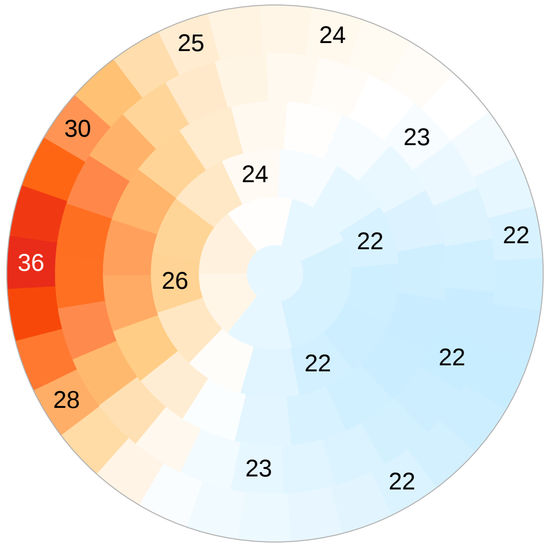

AF Total bilirubin (ľmol/L)

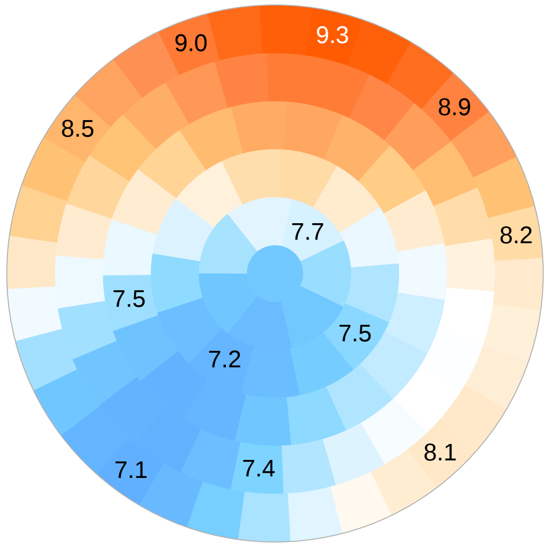

Supplement: Supplementary file 3 — Supplementary Information 3. [file 41598_2022_12198_MOESM3_ESM.pdf]
