# Supplementary material for: Cross-sectional metabolic subgroups and 10-year follow-up of cardiometabolic multimorbidity in the UK Biobank
Source: Sci Rep. 2022 May 21;12:8590. doi: 10.1038/s41598-022-12198-1 (PMC9124207; doi:10.1038/s41598-022-12198-1)
Supplement: Supplementary file 4 — Supplementary Information 4. [file 41598_2022_12198_MOESM4_ESM.docx]

**Supplementary Figure S1** (file: supplementary_figures_S1-S4.pdf)

Participant selection.

**Supplementary Figure S2** (file: supplementary_figures_S1-S4.pdf)

SOM quality control. Sample density indicates the number of UK Biobank participants located within a map district (**A**). Model residuals indicate how well the SOM captures the shape of the metabolic profiles for individuals located within a map district. Values between −3 and +3 are considered acceptable quality (**B**). Data availability indicates the proportion of usable measurement values (**C**). Selected quantitative traits were adjusted for the appropriate drug effects to check if the SOM patterns were confounded by medication (**D-L**).

**Supplementary Figure S3** (file: supplementary_figures_S1-S4.pdf)

Correlation modules of biomarkers. The modules were derived using an agglomerative from the pair-wise Spearman correlation network between 51 metabolic traits. First, edges with R^2^ < 50% were excluded, then an agglomerative spanning tree algorithm was applied to determine highly connected modules.

**Supplementary Figure S4** (file: supplementary_figures_S1-S4.pdf)

SOM colorings for hormones, stratified by sex and the mean age of menopause. Z-scores indicate values of standardized input features as used in the SOM training (three columns of plots on the left). The measured values were not adjusted and are reported in their original measurement units. Furthermore, the map colors are calibrated in such a way that the same numerical value corresponds to the same color in each plot of a specific variable (three columns of plots on the right).

**Supplementary Figure S5** (file: supplementary_figure_S5.pdf)

SOM colorings for men.

**Supplementary Figure S6** (file: supplementary_figure_S6.pdf)

SOM colorings for women.

**Supplementary Table S1** (file: supplementary_tables.xlsx)

Diagnostic codes.

**Supplementary Table S2** (file: supplementary_tables.xlsx)

Cohort characteristics.

**Supplementary Table S3** (file: supplementary_tables.xlsx)

Subgroup profiles.

**Supplementary Table S4** (file: supplementary_tables.xlsx)

Subgroup disease prevalence.

**Supplementary Table S5** (file: supplementary_tables.xlsx)

Subgroup disease incidence.

**Supplementary Table S6** (file: supplementary_tables.xlsx)

Metabolic syndrome.

**Supplementary Table S7** (file: supplementary_tables.xlsx)

Multimorbidity enrichment.

**Supplementary Table S8** (file: supplementary_tables.xlsx)

Multimorbidity overlaps.

**STROBE checklist for cross-sectional studies**

1. Title and Abstract: a) Study design is indicated in the title; b) Main statistical findings are listed in the abstract.
2. Background: Rationale for the study is described in the first paragraph, references to previous scientific findings included in the second and third paragraphs.
3. Objectives: The aims of the study are stated in the last paragraph of Introduction.
4. Study design: Overall study design is included in the title, the unconventional statistical design of the subgrouping analysis is presented in Figure 2 and explained in Methods.
5. Setting: A brief description of the UK Biobank is included in Methods with reference to the main protocol paper by the Biobank investigators. As the UK Biobank is already extensively documented by other authors and a well-known public resource, we did not repeat redundant documentation.
6. Participants: Participant selection is described in Methods and in Supplementary Figure S1.
7. Variables: Disease outcomes were defined according to ICD codes as adjudicated by the UK Biobank and listed in Supplementary Table S1. Age, sex and medications were addressed as potential confounders in Methods.
8. Measurement: The details of the biochemical analyses in the UK Biobank have been published previously and were referenced via the protocol paper.
9. Bias: Age and sex were adjusted when constructed statistical models, as described in the methods. Highly collinear variables were merged as described in the Methods, see also Figure 1.
10. Study size: We used all available data from the UK Biobank with European ancestry.
11. Quantitative variables were used as inputs to the self-organizing map. The data processing is described in Methods and in Figure 2. Subgrouping procedure is described in Figure 3.
12. Statistical methods: a) confounder adjustments are described in paragraphs 3 and 4 in Methods and covariates in regression models in paragraph 6; b) subgrouping framework is described in Methods and first half of Results; c) participants with too few molecular or clinical data were excluded and missingness protocols are included in the software tool; d) population sampling of the UK Biobank has been described previously and referenced in Discussion; e) techniques to assess statistical fluctuations are described in Methods.
13. Participants: Please see Supplementary Figure S1 and first paragraphs in Methods.
14. Descriptive data: Population and subgroup means are extensively documented in Supplementary Tables S2-S7.
15. Outcome data: Exact counts of clinical end-points are listed in Supplementary Tables S2-S7.
16. Main results: a) Unadjusted estimates for disease prevalence and incidence were reported in Figure 5A-F and 6A-F; b) Subgrouping boundaries are visualized in Figure 3; c) both absolute risk for SOM regions and relative risk against reference subgroup were reported.
17. Other analyses: Statistics for multimorbidity and correlation structure were included.
18. Key results: Study aims and the main findings related to them were repeated in the first paragraph of Discussion.
19. Limitations: Discussion includes a paragraph that describes the weaknesses and strengths of the study.
20. Interpretation: Discussion includes a comparison between the subgroup characteristics and previous literature about cardiometabolic risk factors and the role of our findings with respect to the practical relevance and challenges of precision medicine.
21. Generalisability: The representativeness of the UK Biobank has been investigated before and is cited in Discussion, with a caution about generalizing to other ethnicities or populations.
22. Funding bodies are listed in the submission package to the journal.
